# Trends in quality of care and dying perceived by family caregivers of nursing home residents with dementia 2005–2019

**DOI:** 10.1177/02692163211030831

**Published:** 2021-08-28

**Authors:** Maartje S Klapwijk, Sascha R Bolt, Jannie A Boogaard, Maud ten Koppel, Marie-José HE Gijsberts, Carolien van Leussen, B. Anne-Mei The, Judith MM Meijers, Jos MGA Schols, H Roeline W Pasman, Bregje D Onwuteaka-Philipsen, Luc Deliens, Lieve Van den Block, Bart Mertens, Henrica CW de Vet, Monique AA Caljouw, Wilco P Achterberg, Jenny T van der Steen

**Affiliations:** 1 Department of Public Health and Primary Care, Leiden University Medical Center, Leiden, The Netherlands; 2 Huis op de Waard, Marente, Leiden, The Netherlands; 3 Department of Health Services Research, Faculty of Health Medicine and Lifesciences, CAPHRI School for Public Health and Primary Care, Maastricht University, Maastricht, The Netherlands; 4 Living Lab in Ageing and Long-Term Care, Maastricht, The Netherlands; 5 Zorginstituut Nederland, Diemen, North Holland, The Netherlands; 6 End-of-Life Care Research Group, Vrije Universiteit Brussel (VUB) and Ghent University, Brussels, Belgium; 7 Tao of Care, Amsterdam, The Netherlands; 8 Department Sociology, Faculty of Social Sciences, Vrije Universiteit Amsterdam, Amsterdam, The Netherlands; 9 Zuyderland Care, Zuyderland Medical Center, Sittard-Geleen, The Netherlands; 10 Department of Public and Occupational Health, Amsterdam UMC, Locatie VUmc, Amsterdam, The Netherlands; 11 Department of Medical Statistics and Bioinformatics, Leiden University Medical Center, Leiden, The Netherlands; 12 Department of Epidemiology and Data Science, Amsterdam UMC, Location VU University Amsterdam Public Health Research Institute, Amsterdam, The Netherlands; 13 Department of Primary and Community Care, Radboud university medical center, Nijmegen, The Netherlands

**Keywords:** Dementia, geriatrics, palliative care, nursing homes, quality of health care, end-of-life care

## Abstract

**Background:**

Dementia palliative care is increasingly subject of research and practice improvement initiatives.

**Aim:**

To assess any changes over time in the evaluation of quality of care and quality of dying with dementia by family caregivers.

**Design:**

Combined analysis of eight studies with bereaved family caregivers’ evaluations 2005–2019.

**Setting/participants:**

Family caregivers of nursing home residents with dementia in the Netherlands (*n* = 1189) completed the End-of-Life in Dementia Satisfaction With Care (EOLD-SWC; quality of care) and Comfort Assessment in Dying (EOLD-CAD, four subscales; quality of dying) instruments. Changes in scores over time were analysed using mixed models with random effects for season and facility and adjustment for demographics, prospective design and urbanised region.

**Results:**

The mean total EOLD-SWC score was 33.40 (SD 5.08) and increased by 0.148 points per year (95% CI, 0.052–0.244; adjusted 0.170 points 95% CI, 0.055–0.258). The mean total EOLD-CAD score was 30.80 (SD 5.76) and, unadjusted, there was a trend of decreasing quality of dying over time of −0.175 points (95% CI, −0.291 to −0.058) per year increment. With adjustment, the trend was not significant (−0.070 EOLD-CAD total score points, 95% CI, −0.205 to 0.065) and only the EOLD-CAD subscale ‘Well being’ decreased.

**Conclusion:**

We identified divergent trends over 14 years of increased quality of care, while quality of dying did not increase and well-being in dying decreased. Further research is needed on what well-being in dying means to family. Quality improvement requires continued efforts to treat symptoms in dying with dementia.

**What is already known about the topic?**

- Dementia is an incurable condition and in Western countries, most people with dementia die in nursing homes.
- The knowledge base concerning palliative care for people with dementia has expanded.
- Bereaved family caregivers’ experiences and perceptions of the dying phase and the quality of care of their relatives are relevant, as they need to live on with memories.

**What this paper adds?**

- This study shows that from 2005 to 2019, family caregivers increasingly appreciated the quality of care provided to their relative with dementia dying in a nursing home.
- Family caregivers’ evaluation of quality of dying, however, did not improve, indicating that families did not perceive fewer symptoms over time, and even perceived lower well-being while dying (items on serenity, peace and calm).

**Implications for practice, theory or policy?**

- Monitoring trends in the palliative care for people with dementia may aid our understanding of the influence of policy and societal developments.
- Research is needed to explain trends and help to decrease symptom burden and improve quality of dying for people with dementia.

## Introduction

In Western European countries such as the UK and the Netherlands, most people with dementia eventually move to a nursing home, where they reside until death.^1–3^ Nursing home residents may benefit from palliative care with a focus on comfort and quality of life.^4,5^ The cognitive impairment associated with moderate or advanced dementia often leads to limited verbal expression of needs. This complicates the assessment of specific palliative care needs and addressing of symptoms.^6,7^ Family caregivers of people with dementia fulfil important roles as spokespersons, care partners, informants and proxy decision-makers.^8–11^ Their role continues after nursing home admission.^12,13^ Staff should acknowledge the family caregivers’ role in the care for people with dementia, especially at the end of life.^14–16^ Families’ experiences with end-of-life care and their interactions with professional caregivers potentially influence their post-bereavement outcomes.^17^ ‘How people die remains in the memories of those who live on’ is a famous quote in palliative care literature.^18^ Memories of family members reflect the dying experience and may expose specific points for improvement in end-of-life care.^19^ Family caregivers are important judges of the quality of end-of-life care provided to residents with dementia and of their quality of dying.^20,21^ Validated instruments are available to measure quality of care and quality of dying from the family perspective.^22,23^

A small study that investigated data from 2005 to 2010 showed a positive trend in families’ reports of quality of end-of-life care for nursing home residents with dementia.^24^ Exploring such trends can aid our understanding of how the experiences of family caregivers with end-of-life care may have changed, which informs future initiatives to improve palliative and end-of-life care. The present study examines trends in quality of care and quality of dying up to 2019 as judged by family caregivers of residents with dementia in Dutch nursing homes. Various national initiatives aimed to improve knowledge on palliative care in the Netherlands over the last decade. Therefore, the hypothesis is that these trends over a period in which development of dementia palliative care continued, are positive.

## Methods

### Study population

Data from eight studies conducted in the Netherlands in overlapping time windows between 2005 and 2019 were combined for trend analyses (Table 1).^22,24–30^ For seven of the studies, it concerned a secondary analysis of data collected to address various research questions (Supplement). The main goal of the eighth and most recent study was to enhance assessment of trends over time. Some studies employed nationally representative sampling, whereas other studies were regional. All studies collected data retrospectively, and one study also collected data prospectively (Table 1).^22,24–30^ Data collected during any intervention condition were excluded. The data concern 1189 persons with dementia who died in 117 nursing home facilities. One facility contributed to two studies (studies 1 and 3, Table 1). The family caregivers who were the primary contact persons were invited to complete a questionnaire; within 1.5–2 months after death in most studies, and up to about a year after death in two studies (studies 6 and 8, Table 1). All nursing home residents included in these studies received medical care by a certified elderly care physician.^31^

**Table 1.** Overview of datasets combined for trend analyses on quality of care and quality of dying.

Study	Main reference for study/included in early combined analyses	Period	Design	Number of nursing homes, area in Netherlands	Number of residents with dementia (response rate)	Time questionnaire sent to family caregiver after death	Study aim	Timeframe, last	
								EOLD-SWC	EOLD-CAD
1. Gijsberts et al.	*Ned Tijdschr Geneeskd* ^24^	September 2005–June 2007	Retrospective, observational	Four facilities, West/Central	54 (61%)	2 months	Validate Dutch translation.Compare anthroposophical nursing homes to nursing homes without affiliation. Comparison of after-death scores of family caregivers and nurses, and of Dutch and US family caregivers.	Last 90 days	During his/her dying
	*Palliat Med* ^25^								
	*Int Psychogeriatr* ^26^								
2. Van Soest-Poortvliet et al. Psychometric instrument study	*Ned Tijdschr Geneeskd* ^24^	February 2008–April 2009	Retrospective, observational	14 facilities, West/Central	70 (59%)	2 months	Assess psychometric properties of instruments to evaluate quality of care and death in long-term care	Last month	Last week
	*J Am Med Dir Assoc* ^22^								
3. DEOLD Study	*Ned Tijdschr Geneeskd* ^24^	February 2007–July 2010	Prospective and retrospective, observational	40* facilities of 17 health care organisations, nationwide	248 (58%)	6 weeks	Asses factors associated with quality of care and quality of dying	Last week	During his/her dying, only if present
	*Alzheimer Dis Assoc Disord* ^27^								
4. FOLlow-Up Study	*Palliat Med* ^28^	January 2012–June 2014	Retrospective, cluster RCT	18^#^ facilities, nationwide	537 (65%)	6 weeks	Assessment of effect of feedback strategies in perceived end of life care and comfort	Last month	Last week
5. PACE, European study	*J Am Med Dir Assoc* ^29,30^	December 2014–November 2015	Retrospective, six countries also non-dementia, observational	25 facilities, stratified sampling, nationwide	89 (62%)	2–4 months	Comparison of palliative care in nursing homes in six European countries	Last week	Last week
6. Proeftuin Dementie	No publication yet	February 2017–October 2017	Retrospective, observational (intervention not implemented in nursing homes)	Four facilities of one health care organisation, North of NL	16 (43%)	6–13 months	Improving palliative care with mobile palliative care teams	Last week	Last week
7. DEDICATED (Desired Dementia Care Towards End of Life)	No publication yet	February 2018–September 2019	Retrospective, observational	Seven facilities of one health care organisation, South of NL	125 (62%)	6–8 weeks	Improving palliative care for people with dementia and caregivers	Last 3 months	Last week
8. Marente, New data collection	No publication yet	April 2018–December 2018	Retrospective, observational	Six facilities of one health care organisation, West of NL	50 (58%)	3–12 months	Additional data to address research question of possible trend in evaluation end of life care	Last week	Last week

EOLD-SWC: End-of-Life in Dementia-Satisfaction with Care; EOLD-CAD: End-of-Life in Dementia-Comfort Assessment in Dying; DEOLD: Dutch End Of Life in Dementia; FOLlow-up: Feedback on End-of_Life care in dementia; PACE: Palliative Care in care Homes Across Europe; DEDICATED: Desired Dementia Care Towards End of Life.

* Included nursing homes after move.

# Only pre-test and control group in trend analysis.

### Instruments

Quality of end-of-life care was measured with the End-of-Life in Dementia Satisfaction With Care (EOLD-SWC) instrument.^20,23^ It has the most favourable psychometric properties as compared to other such instruments and it comprises 10 items regarding experiences on quality of care from the perspective of the family caregiver.^22^ The items cover decision-making, communication, understanding the resident’s condition and medical care. The response options are: strongly disagree, disagree, agree and strongly agree. Three items are negatively phrased statements, which require reverse coding before summing to total scores that range from 10 to 40. A higher score indicates better quality of end-of-life care.

The End-of-Life in Dementia Comfort Assessment in Dying (EOLD-CAD)^20^ was used to measure quality of dying.^23,32^ The EOLD-CAD comprises 14 items on symptoms such as pain, shortness of breath, choking, and fear. It also includes three positive items in a ‘Well being’ subscale. This subscale consists of items serenity, peace and calm, which require reverse coding. The three response options are: a lot, somewhat and not at all. Total scores range from 14 to 42, a higher score indicating a better perceived quality of dying. Most studies (6 out of 8) referred to the last week of life. One study used a skip pattern for the EOLD-CAD if the relative was not present during dying, setting a higher bar with regard to actual presence to observe comfort (Table 1).

### Cognition

All residents had a physician’s diagnosis of dementia and resided in a psychogeriatric unit. Studies 1, 2, 3 and 5 (Table 1) included staff assessment using the Bedford Alzheimer Nursing Severity-Scale (BANS-S) to measure the severity of the dementia in the months before death. BANS-S scores range from 7 to 28. A score of 17 or higher represents severe dementia.^33,34^ In studies 1 to 5, staff assessed whether residents were fully dependent in eating. Full eating dependence indicates very severe cognitive impairment and is equal to the highest level of impairment on the Cognitive Performance Scale (CPS 6).^35,36^

### Trend analysis

The EOLD-SWC and EOLD-CAD scores in the combined dataset were analysed with mixed models, using time of death relative to the first death in the first study as the independent variable. The models included random effects for season (as Comment 17 seasonality in cause of death might vary between years) and for clustering of residents within nursing homes.^37,38^ In study 7, only the month of death was available due to privacy regulations, and we imputed the 14th for February and the 15th for other months. We provide 95% confidence intervals around the estimate for time. Models were adjusted for characteristics of residents (age and gender), and family caregivers (gender, relationship to resident), region (urbanised Western and central region of the country with greater staffing problems versus other region), and design (prospective enrolment of residents versus retrospectively after death). We conducted sensitivity analyses with additional adjustment for severity of dementia measured with the BANS-S or the eating dependence item (CPS 6) and family caregiver’s age. We also added a quadratic term for time to assess the fit of a non-linear model.

Descriptive statistics were used for respondent characteristics. If less than one third of EOLD items missed, the items were imputed with the patient item mean to generate a total score. All analyses were performed in SPSS Inc, version 25, 2017, IBM, USA.

## Results

The mean age of the residents was 85.5 years; 67.9% were female (Table 2). A little over half (53.7%) had severe dementia and almost a third (29.4%) were fully dependent in eating (no data available for the studies covering 2018 and 2019). Distributions of age, gender and dementia severity were fairly homogeneous between the eight studies (Table 2). Of the family caregivers, the majority were female (62.8%), and most were sons or daughters (in-law) of the resident (65.8%). The EOLD-SWC (quality of care) mean total ranged from 31.9 to 34.1, and the EOLD-CAD (quality of dying) mean total score ranged from 27.2 to 33.3 across studies (Table 3). The correlation between the EOLD-SWC and the EOLD-CAD for quality of dying was weak (+0.27, *p* < 0.001).

**Table 2.** Characteristics of nursing home residents who died with dementia and their relatives.

Mean (SD) or %, [*n*]	Total all studies	Gijsberts	Van Soest	DEOLD	FOLlow-up	PACE	Proeftuin Dementie	DEDICATED	Marente
Number of residents	1189	54	70	248	537	89	16	125	50
Age, mean number of years; (SD) [*n*]	85.5 (7.6) [1178/1189]	85.1 (5.8) [54/54]	88.8 (5.9) [67/70]	85.6 (7.1) [244/248]	84.9 (8.1) [535/537]	85.6 (7.2) [89/89]	85.4 (7.5) [15/16]	85.7 (7.7) [125/125]	85.5 (7.0) [49/50]
Female, % [*n*]	67.9 [807/1189]	80 [43/54]	89 [62/70]	67 [165/248]	68 [366/537]	60 [53/89]	50 [8/16]	60 [74/125]	72 [36/50]
Severity of dementia, BANS-S mean score, (SD), [*n*]	17.1 (4.0) [428/461]	18.6 (3.3) [54/54]	17.9 (4.2) [70/70]	16.3 (3.7) [248/248]	Not available	17.9 (4.9) [56/89]	Not available	Not available	Not available
Severe dementia, BANS-S score 17 or higher % [*n*]	53.7 [230/428]	83 [45/54]	73 [51/70]	41 [102/248]	Not available	57 [32/56]	Not available	Not available	Not available
Full eating dependency (CPS 6), % [*n*]	29.4 [271/923]	33 [16/48]	38 [21/54]	26 [61/237]	29 [155/529]	33 [18/55]	Not available	Not available	Not available
Caregiver female, % [*n*]	62.8 [747/1186]	61 [33/54]	67 [47/70]	61 [151/246]	62 [331/537]	68 [60/88]	63 [10/16]	61 [76/125]	78 [39/50]
Age caregiver, mean number of years (SD) [*n*]	62.0 (11.2) [1126]	Not available	60.6 (8.5) [70/70]	60.6 (11.2) [246/248]	62.7 (11.8) [533/537]	63.4 (11.0) [88/89]	65.3 (9.8) [16/16]	62.4 (10.5) [123/125]	59.8 (9.6) [50/50]
Relationship caregiver, % [*n*]									
Spouse	18.5 [220]	12 [6]	6 [4]	19 [46]	21 [113]	23 [20]	19 [3]	18 [22]	12 [6]
Child	65.8 [782]	71 [37]	87 [61]	66 [161]	63 [338]	60 [53]	50 [8]	70 [87]	74 [37]
Other	15.3 [181]	17 [9]	7 [5]	16 [38]	16 [86]	18 [16]	31 [5]	12 [15]	14 [7]

SD: standard deviation; BANS-S: Bedford Alzheimer Nursing Severity-Scale; CPS: Minimum Data Set Cognitive Performance Scale.

**Table 3.** Total scores for quality of care (EOLD-SWC; *n* = 1169) and quality of dying (EOLD-CAD; *n* = 903) across studies.

Study/project, mean (SD)	EOLD-SWC	*n*/total *n*	EOLD-CAD	*n*/total *n*
1. Gijsberts	31.9 (4.7)	54/54	32.0 (5.4)	52/54
2. Van Soest-Poortvliet	32.1 (5.8)	68/70	30.7 (5.3)	59/70
3. DEOLD	32.6 (5.3)	242/248	33.3 (5.9)	88/90
4. FOLlow-up	34.1 (4.8)	535/537	30.6 (5.6)	466/537
5. PACE	33.8 (5.2)	86/89	29.7 (5.6)	80/89
6. Proeftuin Dementie	30.2 (6.3)	16/16	27.2 (7.2)	13/16
7. DEDICATED	33.7 (5.0)	118/125	30.6 (6.2)	101/125
8. Marente	33.4 (4.8)	50/50	30.8 (5.5)	44/50

EOLD-SWC: End-of-Life in Dementia-Satisfaction with Care; EOLD-CAD: End-of-Life in Dementia-Comfort Assessment in Dying.

**Figure 1.**
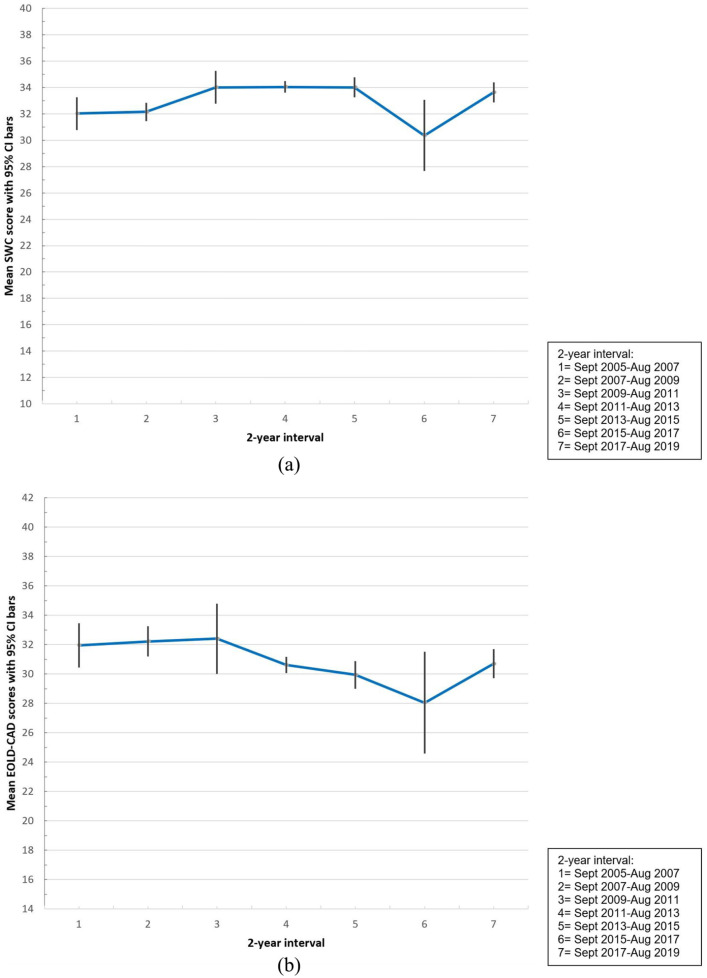
(a) EOLD-SWC means per 2-year intervals with 95% CI bars and (b) EOLD-CAD means per 2-year intervals with 95% CI bars. EOLD-SWC: End-of-Life in Dementia-Satisfaction with Care; EOLD-CAD: End-of-Life in Dementia-Comfort Assessment in Dying; CI: confidence interval.

Figure 1(a) shows unadjusted quality of care means per 2 years; the curve is steeper in earlier years and flattens over time when variable error bars are taken into consideration. The EOLD-SWC total score significantly increased by 0.148 points per year (CI, 0.052–0.244), and in the adjusted model the trend was an additional 0.170 points per year (CI, 0.055–0.285) (Table 4). The EOLD-CAD total score significantly decreased by −0.175 points per year (CI, −0.291 to −0.058; Table 4 and Figure 1(b)) but in the adjusted model the trend was not significant with a decrease of −0.070 points per year (CI, −0.205 to 0.065). The difference of EOLD-CAD with the adjusted model (−0.070 vs −0.175 unadjusted; Table 4) was driven by adjustment for prospective versus retrospective design. The subscale ‘Well Being’ significantly decreased by −0.076 points per year (CI, −0.114 to −0.039) in the unadjusted model, and in the adjusted model by −0.073 points per year (CI, −0.119 to −0.028). The other subscale scores showed no significant trend. Trend models in EOLD-SWC and EOLD-CAD for separate studies are shown in Supplemental Figures S1 and S2.

**Table 4.** Trends in total and item quality of care scores (EOLD-SWC) and in total and subscale quality of dying scores (EOLD-CAD).

	Mean (SD) [*n*]	Trend; coefficient (95%-CI) unadjusted	Trend; coefficient (95%-CI) adjusted
EOLD-SWC total^a^	33.40 (5.08) [1169]	***0.148 (0.052 to 0.244)***	***0.170 (0.055 to 0.285)***
a. I felt fully involved in all decision making	3.41 (0.66) [1171]	***0.017 (0.005 to 0.029)***	***0.017 (0.002 to 0.032)***
b. I would probably have made different decisions if I had had more information	3.30 (0.73) [1137]	0.006 (−0.008 to 0.019)	0.011 (−0.006 to 0.027)
c. All measures were taken to keep my relative comfortable	3.47 (0.66) [1167]	***0.024 (0.012 to 0.037)***	***0.030 (0.016 to 0.045)***
d. The health care team were sensitive to my needs and feelings	3.35 (0.65) [1146]	***0.019 (0.007 to 0.031)***	***0.015 (0.001 to 0.030)***
e. I did not really understand my relative’s condition	3.35 (0.78) [1150]	0.011 (−0.002 to 0.024)	0.010 (−0.005 to 0.026)
f. I always knew which doctor or nurse was in charge of my relative’s care	3.03 (0.78) [1165]	0.014 (−0.000 to 0.029)	0.011 (−0.006 to 0.029)
g. I felt that my relative got all necessary nursing assistance	3.42 (0.66) [1170]	***0.019 (0.007 to 0.031)***	***0.026 (0.011 to 0.040)***
h. I felt that all medication issues were clearly explained to me	3.27 (0.71) [1155]	***0.016 (0.004 to 0.029)***	***0.021 (0.005 to 0.036)***
i. My relative was receiving all treatments or interventions that he or she could benefit from	3.38 (0.66) [1164]	***0.015 (0.004 to 0.027)***	***0.016 (0.002 to 0.030)***
j. I feel that my relative needed better medical care at the end of his or her life	3.42 (0.76) [1159]	0.005 (−0.008 to 0.019)	0.010 (−0.006 to 0.026)
EOLD-CAD total^b^	30.80 (5.76) [903]	−***0.175 (***−***0.291 to*** −***0.058)***	−0.070 (−0.205 to 0.065)
1. Physical distress^c^ (item 1, 2, 3, 4, score range 4–12)	8.34 (2.09) [935]	−0.037 (−0.079 to 0.004)	0.00001 (−0.048 to 0.048)
2. Dying symptoms^d^ (item 4 (part of two subscales), 5, 6, 7, score range 4–12)	8.85 (2.20) [922]	−0.017 (−0.059 to 0.025)	0.017 (−0.032 to 0.067)
3. Emotional distress^e^ (item 8, 9, 10, 11, score range 4–12)	9.54 (2.19) [904]	−***0.061 (***−***0.104 to*** −***0.019)***	−0.026 (−0.077 to 0.025)
4. Well being^f^ (item 12, 13, 14, score range 3–9)	6.14 (1.98) [908]	−***0.076 (***−***0.114 to*** −***0.039)***	−***0.073 (***−***0.119 to*** −***0.028)***

EOLD-SWC: End-of-Life in Dementia-Satisfaction with Care; EOLD-CAD: End-of-Life in Dementia-Comfort Assessment in Dying; SD: standard deviation; CI: confidence interval, italics and bold = *p* < 0.05.

EOLD-SWC item scores are presented because the total score trend is significant.

Cronbach’s α: ^a^EOLD-SWC total: 0.90; ^b^EOLD-CAD total: 0.83; ^c^EOLD-CAD subscale Physical distress: 0.62; ^d^EOLD-CAD subscale Dying symptoms: 0.68; ^e^EOLD-CAD subscale Emotional distress: 0.78; ^f^EOLD-CAD subscale Well being: 0.91.

The sensitivity analyses showed similar estimates. A quadratic term for time was significant for the EOLD-SWC in both the unadjusted (*p* = 0.002) and the adjusted model (*p* < 0.001; Supplemental Figure S3). A quadratic term for change over time was not significant in the unadjusted model for EOLD-CAD (*p* = 0.096) or the adjusted model (*p* = 0.223).

## Discussion

### Main findings

This study investigated trends in family caregivers’ assessments of quality of end-of-life care and quality of dying of nursing home residents with dementia in the Netherlands. From 2005 to 2019, quality of care improved, in particular in the earlier years. Quality of dying did not significantly change in adjusted analyses that included adjustment for prospective design, but scores on the subscale ‘Well being’ nevertheless decreased, also after adjustment.

The statistically significant changes are relevant long-term changes as they may represent ongoing change, and a 2.4 increase in EOLD-SWC total score, for example, nears 3 used in power calculations.^39^ The progressive and terminal nature of dementia and the complex care needs that accompany dementia underpin a palliative approach to care.^5,40^ The evidence-base for palliative dementia care is still small but will expand over the coming years.^41^ The early increase in quality of care in the Netherlands may be related to political developments in palliative care from the late 1990s onward.^42^ A 1997 policy programme aimed to integrate palliative care into the regular healthcare system, to increase practitioners’ skills and knowledge.^43^ In Dutch national dementia plans, however, palliative or end-of-life care is not mentioned.^44^ Treatments for symptom relief in nursing home residents with dementia increased in 2006–2007 compared to the late 1990s.^45^ Reasons for this increase, according to physicians, included growing attention and awareness regarding symptom relief, clearer treatment goals and a focus on quality of life.^45^ Palliative care specialists are consulted for residents in Dutch nursing homes with dementia, in only 2.5% of the cases.^46^ Compared to five other European countries, however, the treating physician in Dutch nursing homes is involved in palliative care more often (in 98.8% of the cases).^30,47^

In the context of increasing quality of end-of-life care as perceived by family and increasing awareness regarding palliative care as perceived by physicians,^45^ finding no improvement on the quality of dying scale and a decline on the ‘Well being’ subscale is counterintuitive. Further, scores on the quality of care items, regarding measures taken to improve comfort and regarding nursing assistance showed the highest increase. Other studies also found weak to moderate associations between quality of care evaluated by families and perceived quality of dying.^48,49^ An interesting artefact may have been introduced by a design issue, with a negative trend for a prospective design (Supplemental Figures S1 and S2). Repeated completion of questionnaires on symptom burden in the prospective study may also have increased family caregivers’ awareness of symptoms in the dying phase. These family caregivers may have been prompted to report more symptoms.

Nevertheless, controlled for design, the trend was also negative for the subscale ‘Well Being’ that comprises the items ‘serenity’, ‘peace’ and ‘calm’. Family caregivers may hold negative perceptions about the end of life with dementia as being undignified, especially in Western societies where autonomy is highly valued.^50^ Increasing media exposure and public campaigns on ‘living well with dementia’, in combination with the Dutch debate on the acceptability of euthanasia in dementia in recent years might influence such perceptions. Lemos Dekker^50^ found that family caregivers of nursing home residents with dementia may feel powerless due to a lack of control over relief of their relatives’ suffering. Higher expectations and standards of care, and increased focus on control and dignity might explain a decrease in their assessment of well-being in dying, while their assessment of concrete symptoms remained unchanged. Future research is needed to disentangle what well-being in dying means to families.

### Strengths and limitations

This study used perspectives from more than 1000 family caregivers of nursing home residents with dementia, over a period of 14 years. It does not evaluate a single reform as there were various initiatives to improve palliative care. Identifying of individual items that did or did not change further enhances the study’s relevance to clinical practice. The EOLD-SWC has strong psychometric properties, whereas there is some ambiguity about the psychometric properties of the EOLD-CAD regarding feasibility, validity and reliability.^22,51^ Although the EOLD-CAD items all assess aspects of quality of dying, the instrument does not cover the full concept of quality of dying, which may include aspects that are more difficult to assess such as retaining identity or dignity.^52^ Nonetheless, other such measures do not perform better or properties are unknown. The EOLD-SWC and EOLD-CAD scales have been widely used after an early comparison of psychometric properties,^22^ which facilitates comparison between countries.^30,53^ This study was limited to the Netherlands, but its EOLD scores are fairly representative for recent European research.^30^ Sample sizes, recruitment methods and the period before death referred to in the EOLD instruments varied between the individual studies in the analyses. There may be residual confounding by unmeasured confounders. However, any confounding by dementia severity is unlikely as adjusted estimated were unchanged in sensitivity analyses.

## Conclusion

This study observed a positive trend in family caregivers’ assessments of the quality of end-of-life care for nursing home residents with dementia over a period of 14 years. Family caregivers’ assessments of quality of dying did not change with regard to symptoms during dying, but according to their assessments the well-being during dying decreased over time. There may be a growing gap between family caregivers’ expectations and actual symptoms and well-being at the end of life. These observations call for further monitoring of quality perceived by family and research to investigate contemporary ideas about what constitutes a ‘good and comfortable death’ at the end of life with dementia.

## Supplemental Material

sj-pdf-1-pmj-10.1177_02692163211030831 – Supplemental material for Trends in quality of care and dying perceived by family caregivers of nursing home residents with dementia 2005–2019 Click here for additional data file. Supplemental material, sj-pdf-1-pmj-10.1177_02692163211030831 for Trends in quality of care and dying perceived by family caregivers of nursing home residents with dementia 2005–2019 by Maartje S Klapwijk, Sascha R Bolt, Jannie A (Nienke) Boogaard, Maud ten Koppel, Marie-José HE Gijsberts, Carolien van Leussen, B. Anne-Mei The, Judith MM Meijers, Jos MGA Schols, H Roeline W Pasman, Bregje D Onwuteaka-Philipsen, Luc Deliens, Lieve Van den Block, Bart Mertens, Henrica CW de Vet, Monique AA Caljouw, Wilco P Achterberg and Jenny van der Steen in Palliative Medicine

sj-pdf-2-pmj-10.1177_02692163211030831 – Supplemental material for Trends in quality of care and dying perceived by family caregivers of nursing home residents with dementia 2005–2019 Click here for additional data file. Supplemental material, sj-pdf-2-pmj-10.1177_02692163211030831 for Trends in quality of care and dying perceived by family caregivers of nursing home residents with dementia 2005–2019 by Maartje S Klapwijk, Sascha R Bolt, Jannie A (Nienke) Boogaard, Maud ten Koppel, Marie-José HE Gijsberts, Carolien van Leussen, B. Anne-Mei The, Judith MM Meijers, Jos MGA Schols, H Roeline W Pasman, Bregje D Onwuteaka-Philipsen, Luc Deliens, Lieve Van den Block, Bart Mertens, Henrica CW de Vet, Monique AA Caljouw, Wilco P Achterberg and Jenny van der Steen in Palliative Medicine
