# Supplementary material for: Trends in quality of care and dying perceived by family caregivers of nursing home residents with dementia 2005–2019
Source: Palliat Med. 2021 Aug 28;35(10):1951–60. doi: 10.1177/02692163211030831 (PMC8637361; doi:10.1177/02692163211030831)
Supplement: sj-pdf-1-pmj-10.1177_02692163211030831 – Supplemental material for Trends in quality of care and dying perceived by family caregivers of nursing home residents with dementia 2005–2019 [file sj-pdf-1-pmj-10.1177_02692163211030831.pdf]

Supplement 1: Description of each study on inclusion of residents and study aims

Study 1 Gijsberts et al. [1-3]

The 54 residents enrolled after death in this study were living in 4 nursing homes in the Western and central regions of the Netherlands. All deaths occurred between September 2005 and June 2007. The first study aim was to translate and validate the End of Life in dementia (EOLD) scales, the second aim was to compare anthroposophical nursing homes with nursing homes without religious or spiritual affiliation on quality of care, dying and symptom burden. The third aim was to compare after-death scores of family caregivers and nurses in the Netherlands and the US. Nurses identified family caregivers who had been most involved in the last months of life of residents diagnosed with dementia. An informed consent form was sent by postal mail 6 weeks after the death of their relative. After receiving consent, a printed questionnaire was mailed and was completed around 2 months after death. The response rate was 61%. The study protocol was approved in 2005 by the Medical Ethics Committee of VU University Medical Center in Amsterdam (number 05/098).

Study 2 Psychometric instrument study, Van Soest-Poortvliet et al. [4]

In this study, 70 residents were included after death. They had resided in 8 nursing homes and had been admitted at least 30 days before death between February 2008 and April 2009. This psychometric properties study was designed to test and compare all available instruments to measure quality of care and quality of death with dementia in long-term care. All residents had a physician's diagnosis of dementia and were admitted to a psychogeriatric unit (units with almost all patients having

dementia) in nursing homes and residential homes. Informed consent was provided by family caregivers. The response rate was 59%. The Medical Ethical Committee of VU University Medical Center, Amsterdam, reviewed the protocol in 2008 as an extension of study 3. (number 06/179, 2006), and provided advice, declaring that the extension also did not fall under the Medical Research Involving Human Subjects Act (WMO).

Study 3 DEOLD= Dutch End Of Life in Dementia, van der Steen et al. [15]

The 491 residents in 19 nursing home organizations covering 34 facilities, enrolled in this study were included using a prospective design in 28 facilities (17 organizations) and a retrospective design in 6 other facilities (2 organizations, after-death questionnaires only). Family after-death questionnaires were returned for 183 residents who resided in the facilities involved in the prospective data collection, and 65 were returned for residents of facilities based on retrospective data collection only. Some residents moved and so the completed after-death questionnaires concerned 6 more facilities, resulting in a total of 40 facilities. Residents with completed after-death questionnaires died between February 2007 and July 2010. The main study aim was to assess factors associated with quality of care and quality of dying. Other aims were to described comfort, symptom burden, pain, decision making and treatment at the end of life. A total of 26 nursing home organizations were invited to participate, 17 agreed, representing a response rate of 65% (2 more nursing home organizations participated after taking the initiative and expressing an interest in participating). The family caregiver received a questionnaire around 2 months after the death of their relative. The response rate was 58%. The study protocol was judged not to fall under the scope of the Medical research Involving Human Subjects

Act (WMO), as declared by the Medical Ethics Committee of VU University Medical Center in Amsterdam, in 2006 *(number 06/179 (2006).*

Study 4 FOLlow-Up= Feedback on End-of Life care in dementia [6]

The 537 residents enrolled in this study all died on a psychogeriatric ward in 18 different nursing homes and were admitted at least 16 days in the last month of life. The time of death was between January 2012 and June 2014. The study aim was to assess the effect of (patient or generic) feedback strategy on perceived end-of-life care and comfort and the study design was a cluster-randomised 3-armed trial. For this trend analysis the residents who resided in the facilities assigned to the control condition (pre-intervention phase and intervention phase) and all residents who died in the pre-intervention period of the intervention groups (patient and generic feedback strategy) were included for analyses. Residents were included if they had stayed in the nursing home at least 16 days in the last month of life and had a dementia diagnosis in their medical file. Around 6 weeks after death the family care giver received an information letter and questionnaire, the response rate was 65%. This cluster-randomised controlled trial is registered in the Netherlands Trial Registration, NL3777 (NTR3942). The research protocol of the Follow-up study was reviewed by the Medical Ethics Review Committee of VU University Medical Center (number 2012/173; judged to not fall under the scope of the Medical research Involving Human Subjects Act (WMO)).

Study 5 PACE= Palliative Care in care Homes Across Europe [7 8]

This study conducted in 6 countries included 329 people of whom 143 were residents of 25 nursing homes on a psychogeriatrics unit (mostly for people with dementia) at

the time of death and 89 family caregivers returned the questionnaires, response rate 62%. These residents died between December 2014 to November 2015. The organizations were invited to participate through a random sampling procedure in each country, based on at least region/province and facility size (number of beds). The aim of the PACE study was to compare palliative care in nursing homes in 6 European countries. The study was funded in the European Commission FP7 program. Consent to participate was obtained when the questionnaire was returned. The Medical Ethics Review Committee of the VU University Medical Center Amsterdam provided a waiver from review as it was judged to not fall under the scope of the Medical Research Involving Human Subjects Act (WMO), 2015.

Study 6 Proeftuin Dementie

This study aimed at setting up palliative care team specialised in palliative care for people with dementia and evaluate it effects in 4 nursing homes. Family caregivers of nursing home residents completed questionnaires before the intervention was pilot tested, and the mobile team was consulted only for home-dwelling persons with dementia. The 16 residents with dementia included in this study (response rate 43%) had been admitted to a psychogeriatric ward of one of four nursing homes in the region where the study was conducted and died between February 2017 and September 2017. Relatives were contacted in March 2018 and asked to complete the questionnaire. Ethical review for the research protocol was provided by the Medical Ethics Review Committee of the Leiden University Medical Center (number (P17.214; judged to not fall under the scope of the Medical research Involving Human Subjects Act (WMO)).

Studie 7 DEDICATED= Desired Dementia Care Towards End of Life

Between February 2018 and September 2019, 206 residents were included in this ongoing end-of-life study. The DEDICATED project aims to improve palliative care for people with dementia. The researchers collect quality of care and quality of dying data over time in one of the participating organizations. The current dataset includes 126 residents who resided on a psychogeriatric unit. For this analysis, we included 125 residents, excluding one resident with a missing date of death from the analysis. The family caregivers in this study received an information letter and questionnaire between 6 to 8 weeks after the residents' death, as part of the routine post-death assessment of the organization. The response rate was 61%. The study protocol was approved by the Medical Ethics Committee of the Maastricht University Medical Center (number METCZ20180026), judged to not fall under the scope of the Medical research Involving Human Subjects Act (WMO).

Study 8 Marente

This study was conceived for the purpose of addressing the research question about trends in family perspectives on quality of care and quality of dying. All caregivers who served as contact persons for staff received questionnaires if their family who resided on a psychogeriatric (mostly dementia) unit and died between first of March 2018 and 31st of December 2018 in one of six facilities of a care organization in the West of the Netherlands. The questionnaires were sent end of March 2019 to 86 relatives and 50 were returned between the first of April and 25th of May 2019, response rate 58%. We enquired with the Medical Ethics Review Committee of the

Leiden University Medical Center; because of use of the same questionnaire as used in study 6, they regarded the ethics review for study 6 as leading and study 8 as an extension with more facilities of the observational part of study 6.

Reference List

1. van der Steen JT, van Soest-Poortvliet MC, Gijsberts MJ, et al. [Improved end-of-life care for patients with dementia: greater family satisfaction and possibly greater end-of-life comfort]. *Nederlands tijdschrift voor geneeskunde* 2013;157(17):A5324. [published Online First: 2013/04/26]
2. van der Steen JT, Gijsberts MJ, Knol DL, et al. Ratings of symptoms and comfort in dementia patients at the end of life: comparison of nurses and families. *Palliative medicine* 2009;23(4):317-24. doi: 10.1177/0269216309103124 [published Online First: 2009/04/07]
3. van der Steen JT, Gijsberts MJ, Muller MT, et al. Evaluations of end of life with dementia by families in Dutch and U.S. nursing homes. *International psychogeriatrics / IPA* 2009;21(2):321-9. doi: 10.1017/s1041610208008399 [published Online First: 2009/02/06]
4. van Soest-Poortvliet MC, van der Steen JT, Zimmerman S, et al. Selecting the best instruments to measure quality of end-of-life care and quality of dying in long term care. *Journal of the American Medical Directors Association* 2013;14(3):179-86. doi: 10.1016/j.jamda.2012.09.019 [published Online First: 2012/11/21]
5. van der Steen JT, Ribbe MW, Deliens L, et al. Retrospective and prospective data collection compared in the Dutch End Of Life in Dementia (DEOLD) study. *Alzheimer disease and associated disorders* 2014;28(1):88-94. doi: 10.1097/WAD.0b013e318293b380 [published Online First: 2013/05/02]
6. Boogaard JA, de Vet HC, van Soest-Poortvliet MC, et al. Effects of two feedback interventions on end-of-life outcomes in nursing home residents with dementia: A cluster-randomized controlled three-armed trial. *Palliative medicine* 2018;32(3):693-702. doi: 10.1177/0269216317750071 [published Online First: 2018/01/19]
7. Van den Block L, Smets T, van Dop N, et al. Comparing Palliative Care in Care Homes Across Europe (PACE): Protocol of a Cross-sectional Study of Deceased Residents in 6 EU Countries. *Journal of the American Medical Directors Association* 2016;17(6):566.e1-7. doi: 10.1016/j.jamda.2016.03.008 [published Online First: 2016/05/11]
8. Pivodic L, Smets T, Van den Noortgate N, et al. Quality of dying and quality of end-of-life care of nursing home residents in six countries: An epidemiological study. *Palliative medicine* 2018:269216318800610. doi: 10.1177/0269216318800610 [published Online First: 2018/10/03]
