# Supplementary material for: Trends in quality of care and dying perceived by family caregivers of nursing home residents with dementia 2005–2019
Source: Palliat Med. 2021 Aug 28;35(10):1951–60. doi: 10.1177/02692163211030831 (PMC8637361; doi:10.1177/02692163211030831)
Supplement: sj-pdf-2-pmj-10.1177_02692163211030831 – Supplemental material for Trends in quality of care and dying perceived by family caregivers of nursing home residents with dementia 2005–2019 [file sj-pdf-2-pmj-10.1177_02692163211030831.pdf]

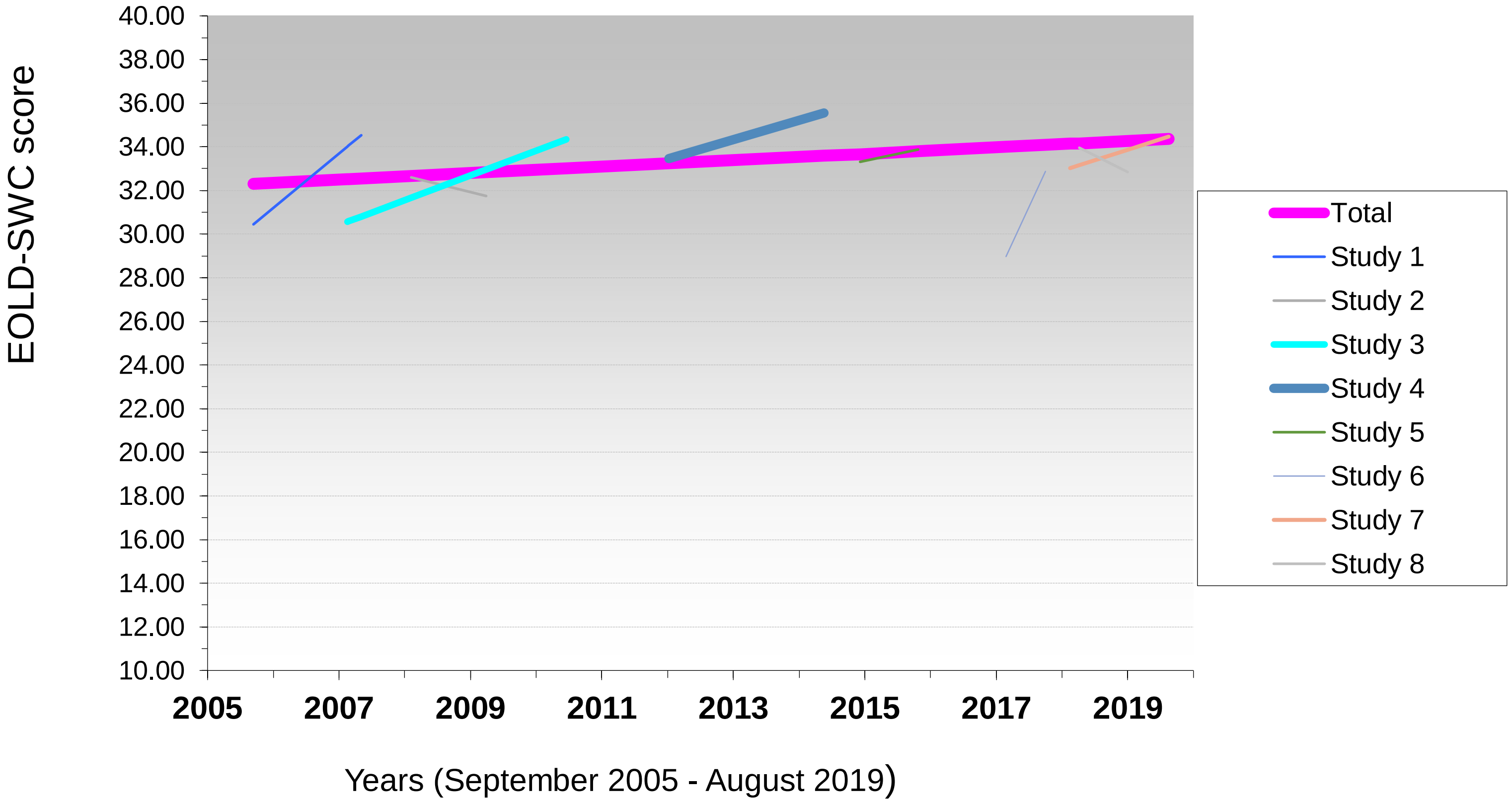

Study 1 Gijsberts et al., n=54

Study 2 Van Soest-Poortvliet et al., n=70

Study 3 DEOLD study, n=248

Study 4 FOLlow-up study, n=537

Study 5 PACE study, n=89

Study 6 Study Proeftuin Dementie, n=16

Study 7 DEDICATED study, n=125

Study 8 Study Marente, n=50

Supplement Figure 1 EOLD-SWC score unadjusted for covariates, with random effects for season and facility

EOLD-SWC= End-of-Life in Dementia Satisfaction with Care, DEOLD=Dutch End Of Life in Dementia, FOLlow-up=Feedback on End-of-Life care in dementia, PACE=Palliative Care in Care Homes Across Europe, DEDICATED=Desired Dementia Care Towards End of Life

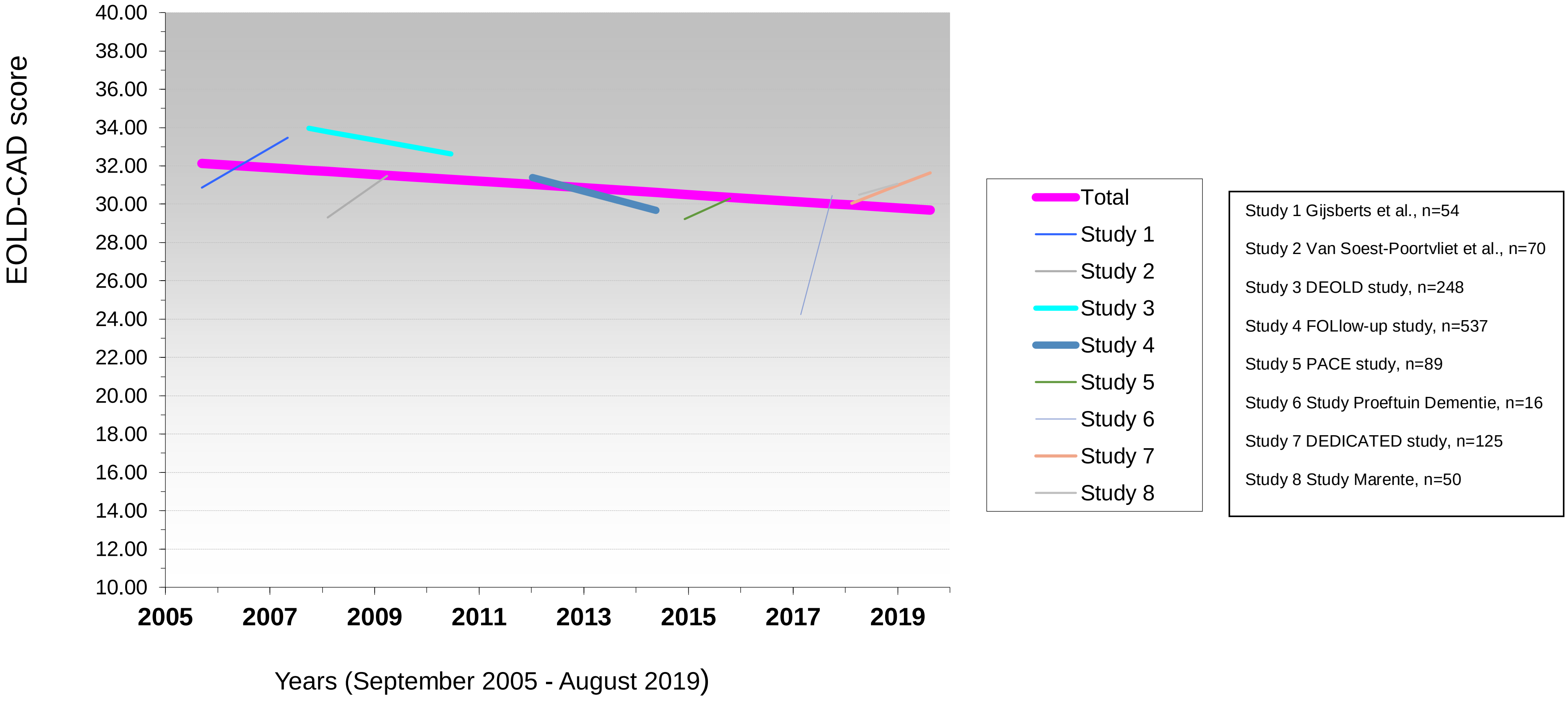

Supplement Figure 2 EOLD-CAD score unadjusted for covariates, with random effects for season and facility

EOLD-CAD=End-of-Life in dementia Comfort Assessment in Dying, DEOLD=Dutch End Of Life in Dementia, FOLlow-up=Feedback on End-Of-Life care in dementia, PACE=Palliative Care in Care Homes Across Europe, DEDICATED=Desired Dementia Care Towards End of Life

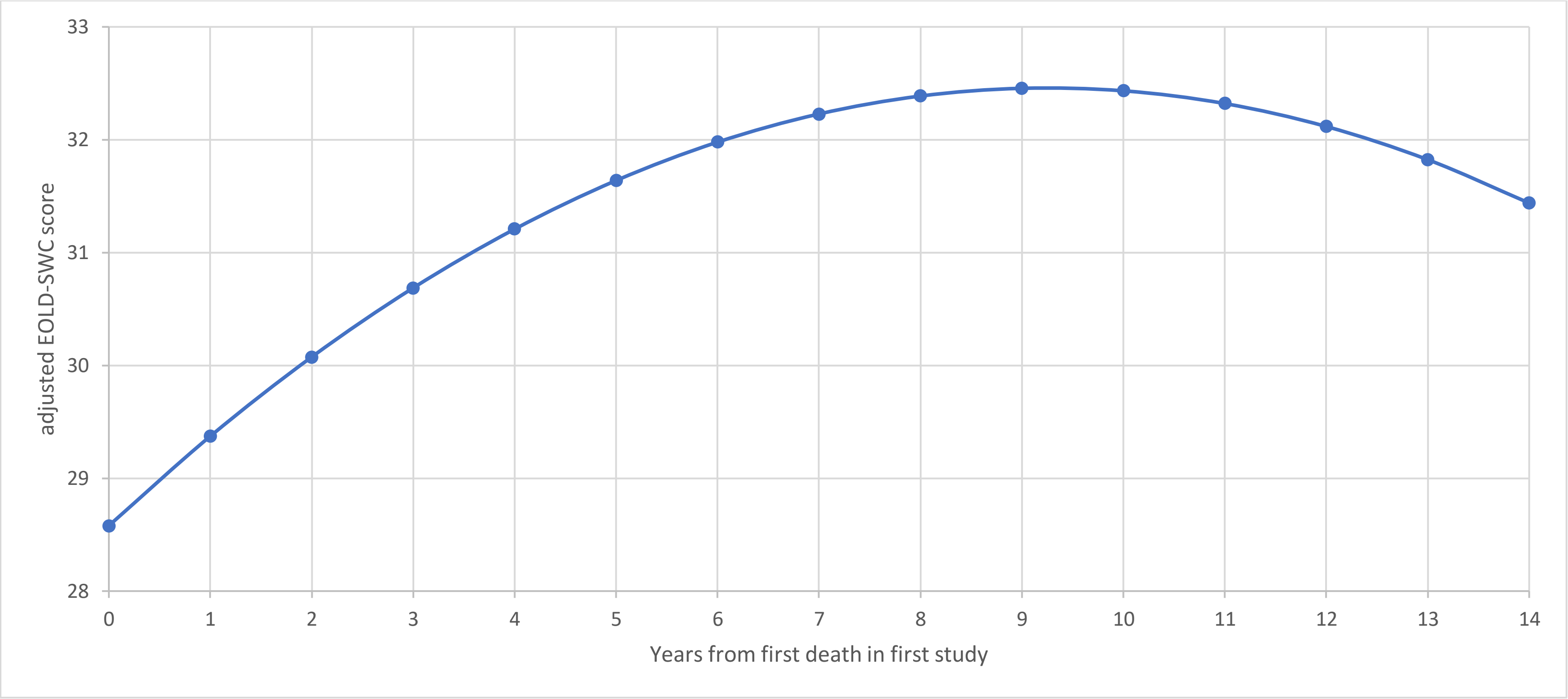

Supplement Figure 3 Quality of care (EOLD-SWC=End-of-Life in Dementia Satisfaction With Care) adjusted model with additional quadratic term for time
